# Modulation of Ion Channels in the Axon: Mechanisms and Function

**DOI:** 10.3389/fncel.2019.00221

**Published:** 2019-05-17

**Authors:** Kenneth J. Burke, Kevin J. Bender

**Affiliations:** Neuroscience Graduate Program and Department of Neurology, Kavli Institute for Fundamental Neuroscience, Weill Institute for Neurosciences, University of California, San Francisco, San Francisco, CA, United States

**Keywords:** presynaptic, action potential, GPCR, modulation, neurotransmission

## Abstract

The axon is responsible for integrating synaptic signals, generating action potentials (APs), propagating those APs to downstream synapses and converting them into patterns of neurotransmitter vesicle release. This process is mediated by a rich assortment of voltage-gated ion channels whose function can be affected on short and long time scales by activity. Moreover, neuromodulators control the activity of these proteins through G-protein coupled receptor signaling cascades. Here, we review cellular mechanisms and signaling pathways involved in axonal ion channel modulation and examine how changes to ion channel function affect AP initiation, AP propagation, and the release of neurotransmitter. We then examine how these mechanisms could modulate synaptic function by focusing on three key features of synaptic information transmission: synaptic strength, synaptic variability, and short-term plasticity. Viewing these cellular mechanisms of neuromodulation from a functional perspective may assist in extending these findings to theories of neural circuit function and its neuromodulation.

## Introduction

**FIGURE 1:**
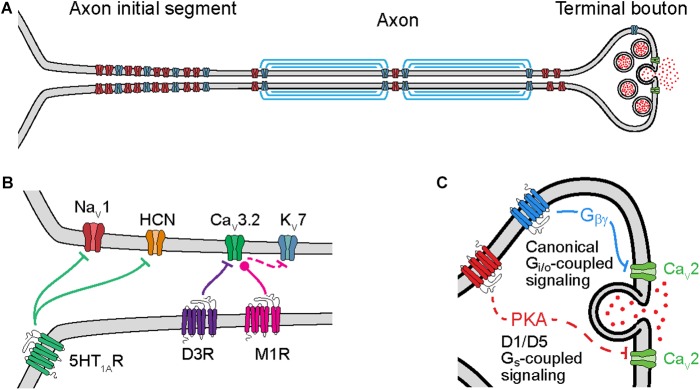
Cellular mechanisms of neuromodulation of axonal ion channels. **(A)** Schematic of axon subcompartments. Sodium (Na_V_), potassium (K_V_), and calcium (Ca_V_) permeable voltage-gated ion channels are shown in red, blue, and green, respectively. **(B)** Schematic of GPCR neuromodulation of voltage-gated ion channels in the axon initial segment. Bars or circles at end of lines indicate a net reduction or increase, respectively, in target channel ion flux as a result of neuromodulation. Note that inhibition of K_V_7 channels is a downstream consequence of Ca_V_3.2 modulation due to changes in intracellular calcium concentration. **(C)** Schematic of GPCR neuromodulation of Ca_V_ in the terminal bouton. Solid lines indicate direct binding of Gβγ subunits to Ca_V_s, dashed lines indicate intermediate steps.

Neuromodulators exert powerful control over both neuronal circuit activity and animal behavior throughout the brain (Marder, 2012; Nadim and Bucher, 2014). Neuromodulatory transmitters engage G-protein coupled receptors (GPCRs), activating intracellular signaling cascades that then can directly activate or modify the properties of ion channels. Neuromodulatory transmitters can bind GPCRs many microns from the site of release, regulating activity within a volume of neuropil (Agnati et al., 1995; Rice and Cragg, 2008; Liu et al., 2018), though cases of more direct synapse-like transmission are also found throughout the brain (Kia et al., 1996; Sesack et al., 2003; Gantz et al., 2013; Courtney and Ford, 2016). Neuromodulatory regulation of ion channels affects how ion channels respond to voltage deflections on short and long time scales, thus affecting how certain features of synaptic input are transformed into neuronal output. This process occurs throughout neuronal arbors, including dendritic and axonal arbors (Athilingam et al., 2017; Labarrera et al., 2018; Yu et al., 2018). Here, we focus on neuromodulation of ion channels in the axon. Recent advances, including the ability to more directly interrogate ion channel function in small axonal compartments, has improved our understanding of how channel function is regulated in these compartments. These modulatory events dramatically affect how synaptic information is integrated to generate patters of action potentials (APs) as well as how those APs are transformed into transmitter release at axon terminals (Figure 1A).

Axonal ion channels are important for many aspects of neuronal function, from the initiation and propagation of APs to the release of neurotransmitter (Figure 1A). APs are initiated in the axon initial segment (AIS), a cellular compartment enriched with voltage-gated ion channels and GPCRs (Figure 1B). At this location, synaptic currents are converted from a graded voltage signal into a train of APs. Due in part to the AP initiation threshold, this transformation is fundamentally non-linear; as a result, the output spike pattern of a neuron is highly sensitive to the neuromodulation of the small fraction of ion channels localized to the AIS.

Neuromodulation of ion channels further down the axon also significantly affects how information is propagated to downstream synapses. Like in the AIS, modulation of ion channels at axonal boutons, the site of neurotransmitter release, also occurs through activation of GPCRs (Figure 1C). Known targets of neuromodulation at the axonal bouton include voltage-gated sodium (Na_V_), potassium (K_V_), and calcium (Ca_V_) channels. In particular, modulation of Ca_V_s can strongly impact the release of neurotransmitters because vesicle release is a direct (but probabilistic and non-linear) function of calcium concentration. Intracellular calcium also influences how the release of neurotransmitter changes with different patterns of presynaptic activity, a critical component of short-term plasticity (STP). As a result, subtle changes to ion channel function at the axonal bouton can result in large effects on synaptic strength, STP, and synaptic information transmission.

In this review, we will provide brief overviews of the biophysical processes involved in AP initiation, propagation, and neurotransmitter release, with an emphasis on how various neuromodulatory mechanisms that target axonal ion channels alter the dynamics of vesicle release and STP at the synapse. We focus primarily on studies performed in vertebrate central circuits, drawing from neuromodulatory systems like dopaminergic and GABA_B_ receptor systems in which significant mechanistic detail has been elucidated. For a recent review of membrane excitability across axonal compartments (see Alpizar et al., 2019). We intend to provide the reader with an intuitive understanding of the connection between neuromodulatory mechanism and function; for more mathematical descriptions of presynaptic release, STP and information transfer, see these references (Tsodyks and Markram, 1997; Dayan and Abbott, 2001; Silver, 2010; Hennig, 2013). By focusing on these biophysical intervention points for neuromodulation and their functional impact, we hope to frame presynaptic neuromodulation in terms that are more easily translated to the study of neuronal circuit dynamics.

## Regulation of Action Potential Initiation

Action potential initiation and propagation are regulated by neuromodulators at several sites. APs are first generated in the AIS, a specialized axonal compartment adjacent to the somatodendritic compartment (Bender and Trussell, 2012; Kole and Stuart, 2012; Huang and Rasband, 2018). The AIS is enriched with sodium and potassium channels that underlie to the rising and falling phases of the AP, as well as other ion channel classes that can augment the AP initiation process, including Ca_V_s and hyperpolarization-activated cyclic nucleotide-gated (HCN) cation channels (Bender and Trussell, 2009; Yu et al., 2010; Martinello et al., 2015; Ko et al., 2016). The precise site of AP initiation can be regulated by activity on short time scales (Scott et al., 2014), and can be structurally modified over longer time scales (Grubb and Burrone, 2010; Kuba et al., 2010). Furthermore, activation of neuromodulatory GPCRs can either enhance or weaken the function of many of these ion channel classes.

Ca_V_3.2 channels, which are expressed in the AIS of many neuronal classes (Bender and Trussell, 2009; Martinello et al., 2015; Clarkson et al., 2017; Dumenieu et al., 2018), are the target of neuromodulation by both dopamine and acetylcholine. These Ca_V_s contribute to subthreshold depolarization and high-frequency AP bursts in many systems (Cain and Snutch, 2013). Dopamine, acting through D3 receptors, hyperpolarizes Ca_V_3.2 voltage-dependent steady state inactivation (Yang et al., 2016). Because the steady-state inactivation level of Ca_V_3.2 channels varies markedly near typical resting membrane potentials (−60 to −90 mV, Serrano et al., 1999; Yang et al., 2016), changes in voltage-dependent inactivation can affect the number of channels available during AP generation. Ultimately, the net effect of dopaminergic modulation is to reduce burstiness, which has been observed in both auditory brainstem and neocortical neurons (Bender et al., 2010, 2012; Clarkson et al., 2017). Cholinergic modulation, by contrast, hyperpolarizes voltage-dependent activation properties of AIS-localized Ca_V_3.2 channels in hippocampal granule cells, increasing AIS calcium concentration near resting membrane potentials (Martinello et al., 2015). The predominant effect of increased basal calcium levels is to reduce calcium-sensitive K_V_7 potassium current, which in turn lowers the threshold for AP initiation. Thus, neuromodulation that affects different biophysical aspects of Ca_V_3.2 function can have bidirectional effects on AP generation.

Axon initial segment HCN channels and sodium channels can also be regulated by neuromodulators, specifically serotonin 5-HT_1A_ receptors. In auditory brainstem, serotonin suppresses HCN channels via G_i/o_-mediated inhibition of cyclic AMP, leading to a hyperpolarization of resting membrane potential and AP threshold (Ko et al., 2016). In mouse cortex and frog spinal cord, serotonin instead has inhibitory effects on axonal sodium channels, reducing sodium channel current density (Cotel et al., 2013; Yin et al., 2015). In cortex, this has a preferential effect on the Na_V_1.2 channel subtype specifically but appears to have a more ubiquitous effect on Na_V_s in the spinal cord. Serotonin’s effects in the spinal cord, however, appear to be due to 5-HT_1A_ receptors expressed in the soma rather than the AIS itself (Cotel et al., 2013). This insight may help explain why physiological effects of serotonin can be observed in the AIS despite uncertainty over the precise subcellular localization of 5-HT1A receptors (Petersen et al., 2017).

## Regulation of Action Potential Propagation and Waveform

Once APs are initiated, they propagate down the axon to sites of vesicle release known as active zones (AZs). This AP propagation itself is not perfectly reliable. For example, APs may fail at axonal branch points (reviewed in Kress and Mennerick, 2009), or near nerve terminals (Kawaguchi and Sakaba, 2015). Furthermore, some spikes generated during high frequency bursts of APs can fail to propagate to release sites (Khaliq and Raman, 2005; Monsivais et al., 2005; Roberts et al., 2008), or exhibit variable conduction velocities that can be augmented in part by dopaminergic regulation of axonal HCN channels (Ballo et al., 2010, 2012). Additionally, astrocytes can regulate AP waveform through ionotropic receptor activation or buffering of extracellular potassium (Menichella et al., 2006; Perea et al., 2007; Bay and Butt, 2012; Bellot-Saez et al., 2017).

Those APs that do arrive at axonal terminals produce a local depolarization that activates Ca_V_s. The resulting calcium influx drives transmitter release via a non-linear reaction dependent on the third- to fourth-order of the local calcium concentration (Katz and Miledi, 1970; Reid et al., 1998; Schneggenburger and Neher, 2000; Scimemi and Diamond, 2012). This non-linear relationship between release and calcium is dependent on several factors, including the physical distance between Ca_V_s and calcium sensors, calcium buffer dynamics and the geometry of the bouton, and the binding affinity and cooperativity requirements of these sensors for calcium (Augustine, 2001; Stanley, 2016; Brunger et al., 2018). Because of this non-linearity, small changes in AP waveform that affect calcium influx can have large effects on release.

Interestingly, however, neuromodulators that regulate AP waveform tend to also have other, more direct mechanisms for influencing *Pr*. For example, dopamine can broaden or narrow APs via D1- or D2-family receptors, respectively, as measured in the primary axon of cortical pyramidal cells (Yang et al., 2013). But the dominant effect of D1 receptors at release sites appears to be direct regulation of Ca_V_ function, rather than AP waveform modulation (Burke et al., 2018). Similarly, while G_i/o_-coupled receptors including the CB1 cannabinoid receptor have been shown to activate G-protein coupled inward rectifier potassium channels (GIRKs) in the axon (Alger et al., 1996; Daniel and Crepel, 2001; Diana and Marty, 2003), the dominant functional effect of G_i/o_-coupled receptor activation at boutons is the direct regulation of Ca_V_s through G_βγ_-dependent signaling (Lüscher et al., 1997; Brown et al., 2004, but see Zurawski et al., 2018), as discussed below.

Action potential waveform adaptation during high-frequency activity has also been repeatedly shown to either suppress or enhance vesicle release, depending on the mechanism of adaptation. In cultured Purkinje cells, attenuation of AP height near terminals strongly suppresses transmitter release (Kawaguchi and Sakaba, 2015). Surprisingly, at hippocampal mossy fiber boutons a similar activity-dependent shortening and widening of axonal APs at high frequency leads to an increase in vesicle release probability (Geiger and Jonas, 2000). The difference between these two synapses may lie in the mechanisms that underlie spike adaptation. AP height is reduced during ongoing activity in both synapses, largely due to reductions in sodium or potassium currents in Purkinje and mossy fiber boutons, respectively. This would be predicted on its own to decrease the activation of Ca_V_s and reduce calcium influx. However, reductions in potassium currents lead to a larger broadening of the AP waveform in mossy fiber boutons than in Purkinje cell axons, increasing the total time the membrane is depolarized. This increased duration of membrane depolarization appears to be sufficient to counteract the reduction in AP height and ultimately increase the overall calcium influx. Indeed, in cerebellar stellate interneurons, a similar widening of presynaptic AP waveforms in response to prolonged somatic depolarization also leads to enhanced transmitter release due to the inactivation of K_V_3 potassium channels (Rowan et al., 2016; Rowan and Christie, 2017). Thus, a subtle mechanistic difference in spike waveform adaptation can lead to the opposite outcome for vesicle release probability.

Axon terminal voltage can also be affected by several mechanisms beyond manipulations of AP waveform, leading to alterations in transmitter release. Terminal Ca_V_s can be sensitive to subthreshold depolarization (Awatramani et al., 2005). This subthreshold activity can be mediated by the local activity of ionotropic receptors, including GABA_A_ receptors which may depolarize axon terminals (Price and Trussell, 2006; Christie et al., 2011), nicotinic acetylcholine receptors (McKay et al., 2007), or glutamatergic receptors (Pinheiro and Mulle, 2008). Alternatively, subthreshold activity at the soma, filtered by axonal cable properties, can propagate to and affect the terminal membrane (Alle and Geiger, 2006; Shu et al., 2006), especially in cells with high input resistance (Christie et al., 2011). This so-called “analog signaling” interacts with APs at the terminal to alter release (Rama et al., 2018). Thus, multiple mechanisms exist to regulate the excitability of the presynaptic bouton and vesicle release, including neuromodulatory GPCR signaling, activity-dependent AP waveform adaptation and subthreshold voltage fluctuations.

## Regulation of Active Zone Voltage-Gated Calcium Channels

Calcium channels localized to active zones are among the most extensively studied effector targets of neuromodulation (reviewed in: Catterall and Few, 2008; Zamponi and Currie, 2013). Typically, presynaptic terminals are enriched with the Ca_V_2 class of calcium channels. This group is comprised of three different isoforms: Ca_V_2.1 (P/Q-type), Ca_V_2.2 (N-type), and Ca_V_2.3 (R-type). Axonal expression of each of these isoforms varies by cell type, and, at times, postsynaptic target (Éltes et al., 2017). For example, some GABAergic synapses express Ca_V_2.1 or Ca_V_2.2 channels exclusively (Poncer et al., 1997; Li et al., 2007; Bender and Trussell, 2009; Cao and Tsien, 2010; Szabo et al., 2014). By contrast, many glutamatergic synapses express a mix of Ca_V_2.1, Ca_V_2.2, and Ca_V_2.3 channels (Brown et al., 2004; Ritzau-Jost et al., 2014). Calcium influx via Ca_V_2.1 and 2.2 channels tends to dominate AP-evoked release (Turner et al., 1992; Wheeler et al., 1994; Bucurenciu et al., 2010; Ritzau-Jost et al., 2014; Burke et al., 2018), whereas 2.3 channels have been shown to be more critical for AP-independent spontaneous release (Ermolyuk et al., 2013). These channels have different kinetics (Colecraft et al., 2001) and exhibit differential activation depending on AP duration. For example, at mossy fiber boutons, Ca_V_2.1 channels are best recruited by fast AP waveforms whereas longer duration waveforms recruit Ca_V_2.1, 2.2, and 2.3 channels to comparable levels (Li et al., 2007). Thus, mechanisms of AP waveform neuromodulation or adaptation discussed earlier may impact synapses differentially depending on the expression levels of different Ca_V_ isoforms. Moreover, isoform-specific mechanisms of neuromodulation could result in synapse-specific changes to short term plasticity (Colecraft et al., 2001). Such changes have been observed with presynaptic forms of long-term potentiation where Ca_V_2.2 channels are preferentially incorporated into synapses following plasticity induction (Ahmed and Siegelbaum, 2009).

Direct inhibition of Ca_V_2 channels by G_βγ_ subunits is perhaps the most common and best understood form of G-protein-mediated neuromodulation of presynaptic calcium channels (reviewed in: Currie, 2010; Padgett and Slesinger, 2010). This form of modulation is common across presynaptically expressed G_i/o_-coupled receptors, including GABA_B_ receptors (Bean, 1989; Mintz and Bean, 1993; Otis and Trussell, 1996; Park and Dunlap, 1998; Takahashi et al., 1998; Chalifoux and Carter, 2011), CB1 cannabinoid receptors (Hoffman and Lupica, 2000; Huang et al., 2001; Kreitzer and Regehr, 2001; Wilson et al., 2001; Zhang and Linden, 2009), type 2 muscarinic acetylcholine receptors (Qin et al., 1997), D2 dopamine receptors (Pisani et al., 2000; Momiyama and Koga, 2001), opioid receptors (Endo and Yawo, 2000; Hjelmstad and Fields, 2003), metabotropic glutamate receptors (Faas et al., 2002), and adenosine receptors (Yawo and Chuhma, 1993; Lüscher et al., 1997). Direct binding of G_βγ_ subunits to Ca_V_2 channels affects channel biophysics by both slowing activation kinetics and by depolarizing the voltage dependence of activation (Bean, 1989; Colecraft et al., 2000). Interestingly, G_βγ_-bound Ca_V_2.2 exhibits a greater number of “reluctant” openings than Ca_V_2.1 channels in which channel open duration is reduced or delayed. Thus, in response to AP depolarizations, the net effect of G_βγ_-mediated inhibition is to reduce calcium influx. However, these calcium currents are also modestly shorter in duration for G_βγ_-bound Ca_V_2.2 channels than Ca_V_2.1 channels (Colecraft et al., 2001). All of these mechanisms converge on reduced calcium influx per AP, which subsequently reduces the probability of neurotransmitter release.

While G_βγ_-dependent signaling is a well-understood form of GPCR-mediated Ca_V_2 channel regulation, GPCRs can also regulate Ca_V_2 channels through other pathways. Dopamine and nociceptin receptors have both been shown to promote channel internalization through GPCR-channel complexes (Altier et al., 2006; Kisilevsky and Zamponi, 2008; Kisilevsky et al., 2008), though whether such mechanisms occur at presynaptic terminals remains unclear (Kisilevsky et al., 2008; Murali et al., 2012). If present at presynaptic terminals, channel internalization could lead to a functional silencing of release sites, essentially reducing synapse number.

D1/D5 dopamine receptors have also been shown to regulate calcium influx through high-voltage activated (presumably Ca_V_2) calcium channels in dissociated striatal neurons via PKA-dependent signaling (Surmeier et al., 1995; Zhang et al., 2002). Similarly, D1/D5 receptors regulate Ca_V_2.1 and Ca_V_2.2 calcium influx in select glutamatergic inputs to prefrontal pyramidal neurons, also via a PKA-dependent pathway (Burke et al., 2018). The precise mechanism of calcium channel modulation remains unclear, but it is clearly distinct from G_βγ_-mediated effects because dopaminergic modulation did not occlude further modulation via GABA_B_ receptors. Moreover, in contrast to G_βγ_-dependent modulation, dopamine did not reduce a single channel’s calcium influx per AP, but rather the probability individual channels would open in response to an AP. This small difference in mechanism resulted in differential functional effects on release; while both dopamine and GABA_B_ reduced *Pr* at this synapse, only GABA_B_ altered STP (further discussed below). This form of presynaptic regulation without marked changes in STP has also been observed for noradrenergic (Delaney et al., 2007) and kappa opioid receptor-dependent modulation (Li et al., 2012; Tejeda et al., 2017). In these cases, the mechanism of action appeared to be either G_βγ_-dependent effects at sites downstream of Ca_V_s (e.g., SNAP25, see Delaney et al., 2007; Zurawski et al., 2018), or via other signaling cascades (e.g., ERK, see Li et al., 2012).

Overall, even when focusing only on ion channel function in axons, there exist many different cellular mechanisms to modulate axon excitability and synaptic transmission. While many of these mechanisms were initially identified due to their functional consequences of regulating vesicle release, we continue to gain insight into how modulation of ion channel biophysical properties affects information transmission across synapses. Further characterization of synaptic transmission before and after neuromodulation will help in identifying the functional role that these mechanisms play *in vivo*.

## Functional Consequences

Neuromodulation of synaptic transmission at the presynaptic axon results in marked changes in information transfer from presynaptic to postsynaptic target. Neuromodulation can affect this process on multiple timescales, affecting three key aspects of transmission at the terminal: (1) synaptic strength, (2) synaptic variability, and (3) STP. By regulating presynaptic calcium influx and vesicle release, axonal neuromodulation can control the strength of specific subsets of synaptic inputs relative to others. But because transmitter release is probabilistic, it also regulates the extent to which individual presynaptic APs are reliably transmitted to the postsynaptic cell. Finally, regulation of STP controls a release site’s strength as a dynamic function of its past activity, which can create a frequency-sensitive postsynaptic signal from a sequence of all-or-none presynaptic APs. Considering synaptic neuromodulation from this functional lens can lead to insight into how cellular mechanisms in the axon might be employed in neural information processing *in vivo*.

### Synaptic Strength and Variability

Neuromodulatory GPCRs can exert fast and reversible control of synaptic strength by altering the probability of neurotransmitter release, *Pr*. In order to focus on broader functional impacts, we will define *Pr* at the level of the synapse, i.e., the probability that the active zone successfully releases a vesicle of neurotransmitter in response to an AP. This quantity is the product of many complex underlying variables, including the number of vesicles available for release, their distance to sources of calcium influx, and the probability of release of that each of these vesicles as a function of intracellular calcium. Within this framework, synaptic strength is therefore defined as the product of *Pr*, the number of release sites *N*, and the quantal size *q* (i.e., the amount of postsynaptic current generated from one vesicle).

**FIGURE 2:**
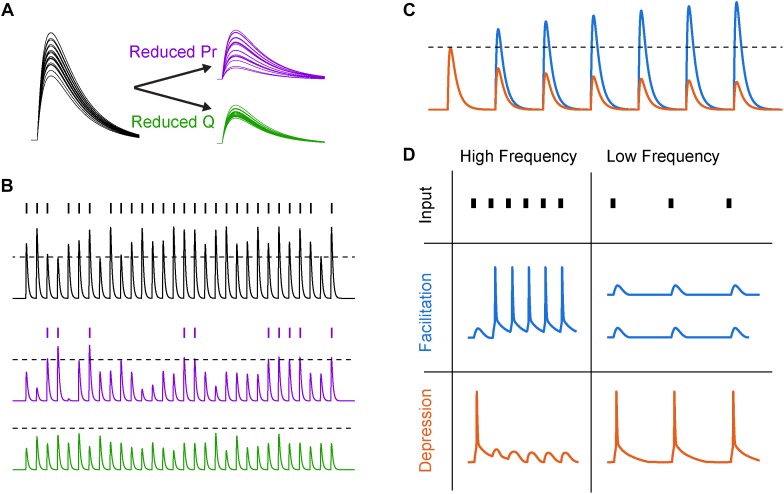
Synaptic variability and short-term plasticity (STP). **(A)** Synaptic variability is differentially affected by presynaptic and postsynaptic neuromodulation. Twenty synaptic potential amplitudes were simulated as a result of a binomial process with Gaussian noise under different conditions. *Left*, under baseline conditions with several release sites and high release probability (*N* = 5, *Pr* = 0.8), synaptic responses are large and reliable. *Right, top*, after reducing release probability by 60%, the trial-to-trial variability increases significantly. *Right, bottom*, reducing postsynaptic charge transfer per vesicle *q* by the same fraction does not lead to an increase in synaptic variability. **(B)** Increased synaptic variability counteracts mean amplitude suppression for simulated presynaptic, but not postsynaptic, mechanisms of neuromodulation. *Top*, a similar binomial process as in **(A)** was used to generate a sequence of simulated synaptic potentials. An arbitrary AP threshold is shown as a dotted line, with the raster plot above indicating crossings of that threshold. *Middle*, after reducing release probability, the arbitrary threshold is still crossed but at a lower rate. *Bottom*, after reducing postsynaptic charge transfer per vesicle, the synapse can no longer cross threshold. **(C)** Schematized subthreshold synaptic potentials in response to a series of presynaptic APs for a facilitating (blue) and depressing (orange) synapse. Dotted line indicates expected synaptic potential amplitude in the absence of STP. **(D)** Responses to high- and low-frequency synaptic input under different STP dynamics. In first row, tick marks indicate timing of presynaptic APs. In second and third row, traces indicate postsynaptic AP generation in response to a strong synapse with facilitating and depressing STP dynamics, respectively.

Changes to both presynaptic release through modification of *Pr* and postsynaptic sensitivity to neurotransmitter *q* will change synaptic strength linearly. Importantly, however, this is only true on average, because presynaptic neuromodulation also changes the *variability* of the synapse, whereas postsynaptic modifications of *q* do not (Figure 2A; for comprehensive review of *Pr* and synaptic variability, see Branco and Staras, 2009). This variability in vesicle release is a major source of overall variability in synaptic transmission, and due to the non-linear relationship between calcium influx and *Pr*, neuromodulators that regulate presynaptic calcium influx will strongly influence both synaptic strength and variance.

While the importance of average synaptic strength in information transmission may be intuitively clear, the role of synaptic variability may be less obvious. Naively, an increase in synaptic variability through reductions in *Pr* might be predicted to make the synaptic signals noisier, degrading the ability of the synapse to convey information about presynaptic AP trains to postsynaptic targets (Zador, 1998; but see Goldman, 2004). However, this may depend on how those synaptic signals are integrated in the postsynaptic cell. For example, in a cell where very few strong input synapses drive APs, reducing *q* below AP threshold could completely abolish postsynaptic spiking, whereas reducing *Pr* would simply reduce the likelihood of AP generation (Figure 2B). If synapses are modeled as simple binomial processes, then the result of reducing *q* is a sharp reduction of spiking near the AP threshold non-linearity, contrasted with a more gradual reduction in postsynaptic AP generation when *Pr* is reduced. In the case of strong *q* suppression (Figure 2B, bottom), the postsynaptic spike sequence then carries *no* information about the sequence of synaptic inputs, whereas in the latter case of modulating *Pr* this information transmission is merely diminished.

This generalization does not, however, extend to all circumstances. First, it ignores non-linearities in dendritic integration, where synchronized excitatory inputs can generate dendritic calcium or NMDA spikes that are either independent of or coordinated with spikes generated in the AIS (Larkum et al., 1999; Schiller et al., 2000; Branco and Häusser, 2011). Whether the neuromodulation of synaptic variability impacts dendritic spikes similarly to how it impacts AIS spikes remains unstudied. Second, for cells that receive many weak synaptic inputs with low *Pr* (e.g., some synapses between neocortical excitatory neurons; Markram et al., 1997), the effects of synaptic variability are more complex and depend on the voltage of the postsynaptic membrane. If the postsynaptic membrane voltage is close to AP threshold, an increase in synaptic variability can push weaker signals over the AP threshold and increase postsynaptic output that is correlated with the weak signal; conversely, if the postsynaptic cell is well above threshold, synaptic noise and increased membrane conductance can lead to reduced output (Shu et al., 2003; Azouz, 2005). In this way, a neuromodulatory weakening of *Pr* and increase in synaptic variability can surprisingly improve the encoding capability of a postsynaptic neuron by broadening its dynamic range (Silver, 2010). Thus, modulation of synaptic strength through modification of release probability can have many indirect effects on neuronal information processing beyond linear changes in synaptic strength.

### Short-Term Plasticity

At many synapses throughout the brain, the strength of a single synapse strength is a function of its previous activity (Figure 2C,D). This process of use-dependent regulation of vesicle release, termed STP, can be altered markedly by neuromodulatory GPCRs. STP depends on many factors, including variable *Pr*, availability of readily releasable vesicles, and AP waveform adaptation with repeated activity. For example, short-term facilitation is mediated through specific isoforms of calcium sensing proteins including synaptotagmin 7, which increase *Pr* in response to relatively low levels of calcium (Jackman et al., 2016). Because the concentration of free calcium in the bouton decays on the timescale of tens of milliseconds (Brenowitz and Regehr, 2007), these proteins allow the calcium from recent APs to non-linearly boost vesicle release over physiologically relevant frequencies of APs. However, the pool of readily releasable neurotransmitter vesicles is finite and depletion of this pool can lead to activity-dependent short-term depression of transmitter release when presynaptic AP rates are high (Betz, 1970; for review see Zucker and Regehr, 2002).

The interplay between these mechanisms of facilitation and depression (and many others) can lead to frequency-dependent filtering of synaptic strength (Tsodyks and Markram, 1997; Dittman et al., 2000). This transformation of presynaptic spike trains into graded, frequency-dependent postsynaptic signals has many functional use cases. For example, facilitating synapses can effectively propagate bursts and minimize low-frequency single APs, which may serve to enhance the signal-to-noise ratio of bursts of activity that encode behaviorally relevant information (Laviolette et al., 2005; Burgos-Robles et al., 2007; Holmes et al., 2012). Conversely, depressing synapses can subtract these bursts from the sequence transmitted to the postsynaptic cell, for example as in adaptation to sensory stimuli (Chance et al., 1998; Chung et al., 2002). Depression has also been shown to implement gain control over inputs with different baseline activity levels, allowing for the postsynaptic cell to respond to relative, rather than absolute, changes in input firing rate (Abbott et al., 1997). Finally, combinations of facilitation and depression can implement band-pass filtering, where activity would be weakened both above and below a characteristic frequency band. Temporal filtering of this sort can enhance responses to transient sensory stimuli (Chance et al., 1998) and may match frequency bands of behaviorally relevant circuit oscillations (Pietersen et al., 2009).

### Canonical Presynaptic Neuromodulation

Short-term plasticity is often regulated in parallel with synaptic strength when a neuromodulator modifies presynaptic calcium influx. For example, changes in calcium influx will affect the calcium sensors that mediate short-term facilitation, but changes in *Pr* will also affect the extent of depletion-mediated short-term depression. In fact, these effects can both occur simultaneously to conceal each other. For example, at high-*Pr* synapses short-term facilitation can be limited by vesicle depletion. At such a synapse, reductions in calcium influx can both reduce *Pr* and unveil underlying facilitation (Zucker and Regehr, 2002; Sakaba, 2006). Indeed, reductions in *Pr* with parallel increases in short-term facilitation as measured by the “paired-pulse ratio” (PPR) are so common that they are considered a hallmark of presynaptic neuromodulation of release (Dittman and Regehr, 1998).

A common example of synaptic suppression with changes in STP is activation of presynaptic GABA_B_ receptors. As described above, GABA_B_ receptor activation suppresses presynaptic calcium currents via G_βγ_-dependent signaling (Mintz and Bean, 1993; Wu and Saggau, 1995). Alongside other calcium-independent mechanisms (Dittman and Regehr, 1996; Sakaba and Neher, 2003), this leads to a potent suppression of release probability while increasing short-term facilitation of subsequent events (Chalifoux and Carter, 2011; Burke et al., 2018). This combination of effects has been hypothesized to support faithful transmission at high frequencies in auditory brainstem (Brenowitz et al., 1998). The behavioral role that this shift in STP plays is unclear; while genetic ablation of the GABA_B_ receptor appears to reduce both anxiety and depressive phenotypes in mice (Mombereau et al., 2004), these mutations likely silence GABA_B_ receptor expression at all subcellular locations. Better tools to disentangle the many cellular functions of the GABA_B_ receptor (including increasing postsynaptic conductance, hyperpolarizing postsynaptic voltage, reducing *Pr*, and increasing facilitation) will be required to attribute behavioral effects to these transformations of synaptic function. Furthermore, tools to precisely separate the two presynaptic effects of GABA_B_ (reducing *Pr* and increasing facilitation) would allow us to better understand the function of other presynaptic neuromodulators that also employ G_βγ_-dependent signaling pathways, such as the CB1 receptor. Alternatively, experiments that compare activation of presynaptic neuromodulators with different effects on synaptic transmission (e.g., two presynaptic inhibitors of *Pr* that differ in effects on facilitation) could more clearly tie these effects on STP to circuit activity and behavior.

### Non-canonical Presynaptic Neuromodulation

While G_βγ_-dependent signaling canonically regulates both synaptic strength and STP, which we will term temporal modulation, some presynaptic neuromodulators appear to regulate *Pr* in isolation, which we will term gain modulation. These latter cases violate the dogma that presynaptic modulation also regulates PPR and demonstrate that temporal and gain modulation do not map cleanly onto presynaptic and postsynaptic mechanisms, respectively. Indeed, any form of modulation that essentially silences synapses (permanently or stochastically) can functionally be characterized as gain modulation. This includes both presynaptic mechanisms (e.g., axonal AP propagation failures, removal of synapses or active zones, slowly dissociating Ca_V_ antagonists) as well as postsynaptic mechanisms (e.g., changes in postsynaptic receptor density or ion channel amplification of synaptic potentials). Similarly, while presynaptic mechanisms to modulate facilitation and depression have been extensively documented, some postsynaptic mechanisms have also been implicated (e.g., local reduction in synaptic driving force; Abrahamsson et al., 2012). Experimental identification of presynaptic or postsynaptic mechanisms therefore require more than simply measuring changes in levels of facilitation or depression (e.g., PPR) and should include other more direct measurements of release probability, such as measurements of synaptic variance (Sigworth, 1980; Saviane and Silver, 2006; Delaney et al., 2007) or optical measurements of transmission at individual synapses (e.g., optical quantal analysis, Oertner et al., 2002; Higley et al., 2009; Little and Carter, 2012; Sylantyev et al., 2013; Boddum et al., 2016; Burke et al., 2018).

One common mechanism for observing presynaptic gain modulation is direct blockade of calcium channels using slowly dissociating antagonists such as cadmium or conotoxin-MVIIC (Hefft et al., 2002; Hjelmstad, 2004; Scimemi and Diamond, 2012). Several neuromodulatory systems have also been found to exert similar effects, including D1/D5 in prefrontal cortex (Gao et al., 2001; Seamans et al., 2001; Burke et al., 2018), D1/D5 at the perforant pathway (Behr et al., 2000), kappa-opioid receptors at amygdalar inputs to the nucleus accumbens (Tejeda et al., 2017), kappa-opioid receptors in the bed nucleus of the stria terminalis (Li et al., 2012), and noradrenergic receptors in central amygdala (Delaney et al., 2007). While these neuromodulators likely employ different signaling pathways, they share in common the regulation of *Pr* apparently without changing STP. Importantly, this presynaptic neuromodulation only of synaptic strength is different from temporal modulation in how it transforms information as it is transmitted across the synapse. While canonical G_βγ_-dependent signaling suppresses transmission from low-frequency presynaptic APs while preserving or enhancing high-frequency transmission, these non-canonical gain neuromodulators that only regulate *Pr* serve to control the average strength of the synapse while preserving the relative strength of transmitted frequencies.

Mechanisms of presynaptic gain modulation can be grouped into two major categories: first, changes in the number of vesicles available for release, and second, changes in release probability per vesicle. An example of this first category is found at the neuromuscular junction of *Drosophila*, where homeostatic adaptation to postsynaptic glutamate receptor blockade is achieved through an enhancement in the size of the readily releasable pool of vesicles (Weyhersmuller et al., 2011). This adaptation is best described as gain modulation because the change in short-term facilitation was significantly smaller than the change in synaptic strength. However, as noted earlier, a finite RRP can lead to vesicle depletion with activity, which leads to frequency-dependent short-term depression (Zucker and Regehr, 2002; Sakaba, 2006). Increasing RRP size could hypothetically remove this depression and unmask facilitation, thus acting as temporal modulation; therefore, increases in RRP size may only implement gain modulation if depletion is not a significant factor under baseline conditions.

An example of the second category, presynaptic gain modulation through changes in *Pr*, was recently observed at excitatory synapses in prefrontal cortex (Burke et al., 2018), and may underlie similar observations made with kappa opioid receptor-dependent modulation in subcortical regions (Li et al., 2012; Tejeda et al., 2017). Here, presynaptic release probability was suppressed by activation of dopaminergic D1 receptors. Unlike some other forms of presynaptic gain modulation, however, the underlying mechanism was a reduction in calcium influx. Interestingly, typical mechanisms that reduce presynaptic calcium, such as a reduction in extracellular calcium concentration, often appear as temporal modulation with strongly correlated with changes in facilitation (Zucker and Regehr, 2002). The gain modulation by the D1 receptor represents an important caveat to this common pattern; because D1 receptor activation suppressed calcium channel open probability, the functional consequence of this modulation was to reduce release probability in a nearly “all-or-none” fashion. Thus, the effect of this modulation bears more resemblance to an AP conduction failure than a “canonical” reduction in AP-evoked calcium channel currents.

### Function Following Form at Nanodomain Synapses

**FIGURE 3:**
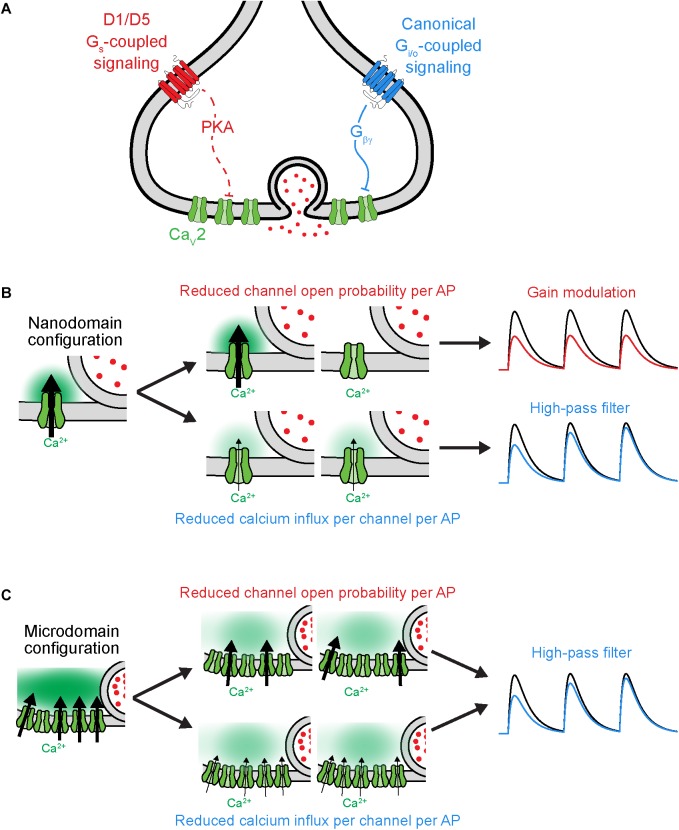
Distinct functional consequences for Ca_V_2 neuromodulation at nanodomain synapses. **(A)** Schematic of GPCR neuromodulation of Ca_V_2 at axonal bouton. **(B)** Functional consequences of different forms of Ca_V_2 regulation under nanodomain coupling between Ca_V_2 and vesicles. *Left*, schematic of nanodomain coupling indicating calcium influx through one channel influencing release. *Center, top*, two possible outcomes resulting from reduced open probability of channel. *Center, bottom*, two outcomes resulting from reducing the current influx through each channel. *Right*, result of two different forms of Ca_V_2 regulation on rate of vesicle release (schematized as postsynaptic potential). Note that the two mechanisms of Ca_V_2 neuromodulation lead to distinct effects under nanodomain coupling. Black and colorized traces indicate baseline transmission and post-Ca_V_2 regulation, respectively. **(C)** As in **(B)**, but under microdomain coupling between Ca_V_2 and vesicles. Note that the two mechanisms of Ca_V_2 neuromodulation lead to similar effects on vesicle release under microdomain coupling. Schematics adapted from Burke et al. (2018).

Why do some neuromodulators that regulate *Pr* also regulate STP, whereas others do not? As mentioned earlier, the size of the RRP and the extent of vesicle depletion could be one explanation. Another is the functional coupling of Ca_V_s and vesicles at active zones (Figure 3). The presynaptic configuration where release of each vesicle is driven by calcium influx through many Ca_V_s is termed a calcium *microdomain*; when release is driven by very few Ca_V_s per vesicle, this is termed a calcium *nanodomain* (for review, see Stanley, 2016). Regulation of the probability of individual Ca_V_ channel opening has been shown to lead to gain and temporal modulation in nanodomain and microdomain configurations, respectively; by contrast, Ca_V_ calcium influx per AP can lead to temporal modulation under both configurations (Figure 3B,C; Otis and Trussell, 1996; Hefft et al., 2002; Hjelmstad, 2004; Eggermann et al., 2012; Scimemi and Diamond, 2012; Burke et al., 2018). Thus, at synapses with nanodomain coupling between Ca_V_s and vesicles, the precise mechanism by which Ca_V_s are modulated can dictate whether a neuromodulator causes gain or temporal modulation. In other words, any observations made where a neuromodulator employs a mix of apparent pre- and postsynaptic mechanisms (e.g., an increase in CV without an increase in PPR) should be couched aside experiments designed to assess whether the same synapses utilize nano- or microdomain release mechanisms.

The neuromodulation of synaptic strength has been understudied in the context of functional coupling between Ca_V_s and vesicles. The aforementioned experiments examining D1 receptor modulation in PFC suggested a critical role of Ca_V_-vesicle functional coupling in its effects on synaptic transmission (Burke et al., 2018). These synapses appear to employ a nanodomain release configuration in which differential Ca_V_ recruitment per AP affects STP (Scimemi and Diamond, 2012). At these synapses in PFC, D1 receptor activation reduced individual Ca_V_ channel open probability at the presynaptic axon, leading to gain modulation of synaptic transmission, whereas activation of the GABA_B_ receptor reduced Ca_V_ current per AP and led to temporal modulation. Taken together, these data led to the hypothesis that D1 activation would modulate postsynaptic responses to all input frequencies similarly, whereas GABA_B_ activation would preferentially suppress responses to low-frequency inputs. Experiments quantifying postsynaptic AP generation confirmed this hypothesis. Whether this functional distinction between gain and temporal modulation extends to naturalistic patterns of activity seen *in vivo*, and what functional role it may play in circuit neuromodulation, has yet to be investigated.

## Future Directions

While the use of fixed frequency synaptic stimulation is both common and important in investigations of cellular mechanisms of synaptic transmission, it also likely obscures how STP and its neuromodulation affect the noisy and irregular patterns of active neurons *in vivo*. Similarly, many effects of neuromodulation observed in *ex vivo* acute slice preparations have yet to be linked to specific behavioral outcomes *in vivo*. Recent work has begun to investigate how different synaptic mechanisms contribute to neuronal computations *in vivo* (Bolding and Franks, 2018; Evans et al., 2018; Lien and Scanziani, 2018). As the experimental technology to measure neuronal activity *in vivo* continues to improve, it is becoming increasingly possible to bridge the gap between mechanistic studies *in vitro* and functional studies *in vivo* and better incorporate synaptic neuromodulation into theories of neuronal circuit function. The highly non-linear behavior of synapses, including their variability, short-term dynamics and complex neuromodulation, are likely to be a critical component of future research into how cellular processes impact neural circuits and behavior. It will be critical to move from mere identification of synaptic connectivity to the identification of the neural codes employed by these connections, as well as identification of when, where, and how neuromodulators are engaged *in vivo*. To achieve this, new tools that can provide real-time measurements of neuromodulatory activity, including sensors for neurotransmitters (Jing et al., 2018; Marvin et al., 2018; Patriarchi et al., 2018; Sun et al., 2018) and intracellular signaling molecules (Violin et al., 2003; Jiang et al., 2017; Ma et al., 2018), will be critical. These tools can be complemented by methods to measure and manipulate activity patterns at individual neurons and even individual synapses (Lee et al., 2019), including both genetically and functionally defined neural circuits (Guenthner et al., 2013; Grewe et al., 2017; Wang et al., 2017; Parker et al., 2018). Together, these technological advances will allow us to quantify how neuromodulators regulate complex activity patterns as they propagate throughout synaptic networks. Moreover, more precise tools are needed to dissect the relative contributions of pre- and post-synaptic effects of neuromodulatory GPCRs, for example distinguishing the functional roles of GABA_B_ activation in changing postsynaptic conductance versus presynaptic *Pr* and STP (Zurawski et al., 2018). The ability to disentangle these many distinct effects will be critical in developing a quantifiable and falsifiable theory of the role neuromodulation plays in synaptic computation.

## Author Contributions

All authors listed have made a substantial, direct and intellectual contribution to the work, and approved it for publication.

## Conflict of Interest Statement

The authors declare that the research was conducted in the absence of any commercial or financial relationships that could be construed as a potential conflict of interest.
